# Home Department

**Published:** 1886-04

**Authors:** 


### HOME DEPARTMENT.

**Beans Baked.**—Soak a pint of white beans over night; pour off the water in the morning and boil the beans in salted water until they are mealy. Then put them in an earthen pudding dish; add a coffee cupful of rich cream and a tablespoonful of sugsr, and bake in a moderate oven until brown. These are more delicate than the beans baked with pork.

**Delicious Little Fried Cakes.**—Beat two eggs well, add to them one ounce of sifted sugar, two ounces of warmed butter, two tablespoonfuls of yeast, a teacupful of lukewarm milk and a little salt. Whip all well together, then stir in by degrees one pound of flour, and, if requisite, more milk, making thin dough. Beat it until it falls from the spoon, then set it to rise. When it has risen, make butter or lard hot in a frying pan, cut from the light dough little pieces the size of a walnut, and without moulding or kneading, fry them pale brown. As they are done, lay them on a napkin to absorb any of the fat.

**Cocoanut Bread.**—The safest way for invalids is to soak the grated nut in a pint of cold water for an hour and a quarter, then put the mixture in a farina-kettle, warm to blood heat, and set it back onto the stove where it will keep warm for about two hours. Then bring it nearly to a boil, removing the kettle from just before the cocoanut water begins to bubble. Let it stand until entirely cold, strain out the nut and use the water for mixing. Add the milk of the nut if it is entirely sweet. Grate the whole of the cocoanut, and add enough water to cover it before soaking and heating; when you have strained out the nut and add its milk, use as much flour as the fiuid will wet to the proper consistency.

**Graham Biscuit.**—Mix Graham flour with cold water, forming dough just stiff enough not to stick to the moulding board; if the flour is of red wheat, or country ground, it must be sifted and cold the white wheat is generally purer, and better for sifting. Knead very thoroughly, as for "beat biscuit," until it is smooth and elastic. Then form into rolls three or four inches long, and three quarters of an inch thick, without any dry flour sticking to them. Make them out rapidly and place a little apart in a pan; prick well with a fork, and put them in the oven it must be hot enough to brown nicely. Bake about half an hour; when done they will not yield to pressure between the thumb and finger. When taken from the fire spread them on the table to cool unless you wish to eat them warm. They are good both cold or warm.

**Lemon Pudding in New Styles**.—Cream up one tablespoonful of fresh butter with one teacupful of sugar. Two eggs must be beaten up very light, and two lemons provided for seasoning. Dissolve a tablespoonful of corn-starch in a little cold milk, then pour over it a teacupful of boiling water. Add to this the butter, sugar and eggs. Rub the lemons until soft, grate the rind into the batter, and also strain into it the juice. Bake in puff paste. This quantity makes two puddings, and it is an excellent dessert.

**Cranberry Sauce**.—Now is the season of cranberries in nearly every city household. The best way to cook them is not so generally known as is the berry. Lucy Linand gives, in the *Pacific Rural Press*, this very fine manner of making an attractive dish of cranberries. To a pound of cranberries we allow one pound of white sugar. Dissolve the sugar in a very little water, boil it ten minutes, making a rich syrup, from which all scum must be removed. Have the cranberries well washed and picked over, put them in the syrup and boil until they are quite soft and of a fine color. Dip two small fancy moulds into cold water for a second, then fill with the cranberries to the top. Next day, or when cold, turn out upside down upon some pretty glass plates, and a much more ornamental dish will be the result than if the cranberries are coooked and served the usual way.

A salad of some sort gives a piquancy to a well-ordered dinner, and by many people is considered a necessary adjunct to a perfect meal. At the head of this list stands the time honored.

### THE OLD PLAN AND THE NEW.

Mr. James F. Morse, Vice-President of the Security Mutual Benefit Society of New York, No. 233 Broadway, has recently placed insurance to the amount of $100,000 on the lives of ex-Senator Arkell and his son, W. J Arkell, proprietor of the Albany *Evening Journal* and the *Judge*. This insurance has been placed in the above named and other leading companies doing business on the assessment plan. The annual cost of carrying it will be about $600. In the old life or level premium companies the cost would be six or seven times as much annually. The Arkells are among the leading business men of the country, and their endorsement of this method of life insurance will carry weight in the business community.—*New York World.*

We are informed that the exact annual cost of carrying the above amount of insurance on the old plan would be *four thousaud five hundred dollars*. The Security Mutual Benefit Society was incorporated in 1881, and the average cost for assessments to a member forty years of age has been less than five dollars a year for each one thousand dollars of insurance.

### RECENT PUBLICATIONS.

**The Century** for April is like its predecessors, most excellent. Three anecdotal articles in tnis number form a most entertaining account of the famous Confederate cruiser *Alabama* and her duel with the *Kearsarge*. P. D. Haywood, who was a seaman on the *Alabama*, describes "Life on the *Alabama*," with graphic humor; Lt. Commander John McIntosh Kell, in his paper, gives an authoritative account of the reasons which impelled Captain Semmes to try issues with the *Kearsarge*, and of the incidents of the fight; while Surgeon John M. Browne of the *Kearsarge* contributes the Union history of that stirring event. In point both of illustrations and anecdotal interest, these articles are perhaps second to none that have appeared in the Century War Series. In "Memoranda on the Civil War," Captain Charles King replies to General Pope, in vindication of his father, General Rufus King. Professor John J. Tigert makes a suggestion in regard to "Government Aid in the Marking of Battle-Fields"; and Colonel L. B. Northrop, the Confederate Commissary-General, rcplies to criticisms by Generals J. E. Johnston, Beauregard, and Imboden. The other articles are all of great interest but we cannot find room to mention them. The only way to get a full idea of the magazine, is to buy and read it—a thing which all of good taste and lover of books is sure to do.

THE DISEASES OF THE SKIN Their description, diagnosis and treatment. By Balmanno Squire, M. B., London, Surgeon to the British Hospital for diseases of the skin, London, Eng. 16mo. Cloth. 194 pages. Price, $1.

This is a comprehensive treatise, in a condnsed form, on the wide department of medical science that is embraced by it.

The type and size of paper have been so arranged as to bring the work down to the compass of a hand-book or manual and to permit of its propuction at a moderate price. 16mo. Cloth. 194 pages.

For sale by Booksellers generally, or mailed, postpaid, on receipt of price by A. N. Marquis & Co., Publishers, Lakeside Building, Clark and Adams Sts., Chicago, Ill.

